# Elastogenesis Correlates With Pigment Production in Murine Aortic Valve Leaflets

**DOI:** 10.3389/fcvm.2021.678401

**Published:** 2021-06-22

**Authors:** Joshua D. Hutcheson, Florian Schlotter, Michael D. Creager, Xiaoshuang Li, Tan Pham, Payal Vyas, Hideyuki Higashi, Simon C. Body, Masanori Aikawa, Sasha A. Singh, Lidia Kos, Elena Aikawa

**Affiliations:** ^1^Department of Biomedical Engineering, Florida International University, Miami, FL, United States; ^2^Biomolecular Sciences Institute, Florida International University, Miami, FL, United States; ^3^Center for Interdisciplinary Cardiovascular Sciences, Division of Cardiovascular Medicine, Department of Medicine, Brigham and Women's Hospital, Harvard Medical School, Boston, MA, United States; ^4^Heart Center Leipzig at Leipzig University, Department of Internal Medicine/Cardiology, Leipzig, Germany; ^5^Department of Biological Sciences, Florida International University, Miami, FL, United States; ^6^Center for Perioperative Genomics, Department of Anesthesiology, Perioperative, and Pain Medicine, Brigham and Women's Hospital, Harvard Medical School, Boston, MA, United States; ^7^Cardiovascular Division, Department of Medicine, Center for Excellence in Vascular Biology, Brigham and Women's Hospital, Harvard Medical School, Boston, MA, United States; ^8^Department of Human Pathology, Sechenov First Moscow State Medical University, Moscow, Russia

**Keywords:** aortic valve, melanocytes, elastin, structure, neurons, glia

## Abstract

**Objective:** Aortic valve (AV) leaflets rely on a precise extracellular matrix (ECM) microarchitecture for appropriate biomechanical performance. The ECM structure is maintained by valvular interstitial cells (VICs), which reside within the leaflets. The presence of pigment produced by a melanocytic population of VICs in mice with dark coats has been generally regarded as a nuisance, as it interferes with histological analysis of the AV leaflets. However, our previous studies have shown that the presence of pigment correlates with increased mechanical stiffness within the leaflets as measured by nanoindentation analyses. In the current study, we seek to better characterize the phenotype of understudied melanocytic VICs, explore the role of these VICs in ECM patterning, and assess the presence of these VICs in human aortic valve tissues.

**Approach and Results:** Immunofluorescence and immunohistochemistry revealed that melanocytes within murine AV leaflets express phenotypic markers of either neuronal or glial cells. These VIC subpopulations exhibited regional patterns that corresponded to the distribution of elastin and glycosaminoglycan ECM proteins, respectively. VICs with neuronal and glial phenotypes were also found in human AV leaflets and showed ECM associations similar to those observed in murine leaflets. A subset of VICs within human AV leaflets also expressed dopachrome tautomerase, a common melanocyte marker. A spontaneous mouse mutant with no aortic valve pigmentation lacked elastic fibers and had reduced elastin gene expression within AV leaflets. A hyperpigmented transgenic mouse exhibited increased AV leaflet elastic fibers and elastin gene expression.

**Conclusions:** Melanocytic VIC subpopulations appear critical for appropriate elastogenesis in mouse AVs, providing new insight into the regulation of AV ECM homeostasis. The identification of a similar VIC population in human AVs suggests conservation across species.

## Introduction

The aortic valve (AV) controls unidirectional blood flow from the left ventricle of the heart to the aorta for systemic circulation (1). Both congenital aortic valve malformations and adult onset aortic valve disease significantly contribute to cardiovascular morbidity, but an incomplete understanding of the factors that govern valve function, including the active cellular contributions to valvular homeostasis, has hindered the development of treatment options (2). A precise ECM microarchitecture of the AV leaflets dictates appropriate valvular function (3). Human AV leaflets consist of three distinct ECM layers, the collagen-rich fibrosa, glycosaminoglycan (GAG)-rich spongiosa, and elastin-rich ventricularis (4). A specialized cell population known as valvular interstitial cells (VICs) resides within valve leaflets and maintains the ECM (5), but VIC heterogeneity has confounded valvular research. No previous reports exist on the role of VIC subpopulations in the production of specific ECM components within the aortic valve microarchitecture.

Studies of normal and diseased AVs have identified VICs with markers of smooth muscle cells, fibroblasts, myofibroblasts, and osteoblasts (6–8). Although melanocytes are most widely studied for their role in controlling skin and eye pigment, melanocytic cells are also observed in mammalian cardiovascular tissues. Notably, pigmented melanocytes appear in the AV leaflets of C57BL/6 mice commonly used in laboratory research (9), complicating histological analyses due to the associated dark pigmentation that can confound the assessment of dark dyes (e.g., von Kossa). These cells have been generally ignored regarding potential valvular function; however, pigmented regions of tissue exhibit increased mechanical stiffness compared to nonpigmented regions in C57BL/6 mouse valves (10), suggesting that the underlying melanocytes may contribute to valve biomechanics. The lack of research interest in these cells may be attributable to a lack of evidence demonstrating the existence of melanocytes in human AV leaflets.

Melanocytes share developmental precursors with neuronal and glial cells (11, 12). Previous histological studies have suggested innervation of AV leaflets (13). A study of neural function in porcine aortic roots showed that specific activity of aortic valve neurons can modulate leaflet contraction (13). We recently reported the presence of glial markers in human AV leaflets (14). The contribution of neuronal and glial cells to AV maintenance and function, however, remains unknown. Here, we show that AV cells that exhibit melanocytic markers also exhibit phenotypic markers of neuronal and glial cells. Genetic mutations that alter pigmentation leads to phenotypic changes in AV elastic fibers. Furthermore, we demonstrate that neuronal and glial VICs within human AV leaflets express a melanocyte phenotypic marker. Human VICs associate with similar ECM components as their murine counterparts, suggesting that melanocytic VICs may contribute to AV homeostasis across species. Therefore, consideration of these cells should be given in studies of AV development and disease.

## Methods

### Procurement and Isolation of AV Tissues

AV leaflets were collected from AV replacement surgeries for severe AV stenosis [Brigham and Women's Hospital (BWH) IRB protocol number 2011P001703]. Non-diseased AVs were obtained from autopsy (BWH IRB 2014P001505) after exclusion of the presence of cardiovascular pathology. The AV samples were handled in phosphate-buffered saline (PBS) on ice and washed in PBS trice, and 1.5-mm slices covering the base and the tip of the AV were embedded in optimum cutting temperature compound (OCT, Sakura Finetek USA, Torrance, CA, USA).

### Mice and Valve Melanocyte Labeling

All animals used in this study were housed in the Animal Care Facility at Florida International University (Miami, FL, USA). Animal work was performed according to institutional guidelines established by the National Institutes of Health. Wild-type (C57BL/6/J) and *Kit*^*Wv*/*Wv*^ were originally obtained from The Jackson Laboratory (Bar Harbor, ME, USA). K5*-tTA;TRE-Edn3* (for simplicity, K5-Edn3) transgenic mice were generated in the laboratory of Dr. L. Kos as described in Garcia et al. (15). The K5-*Edn3* skin and coat are dark compared to nontransgenic littermates eliminating the need for genotyping.

To genetically label tyrosinase-expressing cells in the murine AV, we crossed TYR-CreERT2 with the reporter mT/mG mice resulting in the offspring TYR-CreERT2/ mT/mG mice. TYR-CreERT2 mice (16) have expression of CreERT2 fusion protein directed by mouse tyrosinase (Tyr) promoter/enhancer regions. When bred with mice containing loxp-flanked sequences, 4-hydroxytamoxifen (4HT)-inducible Cre-mediated recombination results in the deletion of the floxed sequences in the Tyr-expressing cells of the offspring. In mT/mG mice (17), Cre-mediated recombined cells (those that express tyrosinase) are labeled with green fluorescent protein (GFP), while the nonrecombined cells are labeled with tdTomato. The membrane-targeted tdTomato and GFP fluorescent proteins in mT/mG mice allow the labeling to be visualized at single-cell resolution. The labeling of Tyr-expressing cells in the leaflets of adult TYR-CreERT2/mT/mG mice was performed by intraperitoneal injection of 1 mg tamoxifen (Sigma-Aldrich, St. Louis, MO) dissolved in peanut oil for 5 consecutive days. The hearts of the mice were collected at least 3 days after the last tamoxifen injection for two-photon imaging. After tamoxifen induction, Tyr-positive Cre recombinase expressing cells show cell membrane-localized green GFP (mG) fluorescence expression replacing the red dTomato (mT) fluorescence.

### Histological Analyses and Quantification

Murine (*N* = 3) and human AVs (*N* = 4) were embedded in frozen OCT compound. Murine samples were either embedded ventricularis side down or were cut between their noncoronary and left-coronary leaflets, extended and embedded perpendicularly so as to obtain longitudinal sections. Human AV samples were embedded perpendicularly, also so as to obtain longitudinal sections for easier comparison with mouse sections. Seven-micrometer sections were cut using Cryostat (Leica CM3050 S, Buffalo Grove, IL, USA). Cryosections undergoing Movat's pentachrome staining and van Gieson staining were dried and then fixed in a 10% formalin solution. Sections were washed in distilled water followed by two washes in PBS. Protocols included with the staining kits (Russel-Movat Pentachrome Kit, American MasterTech KTRMP, Lodi, CA, USA; Richard-Allan Scientific Elastic Stain, ThermoFisher Scientific 87017, Waltham, MA, USA) were then followed for each stain.

Sections that underwent immunohistochemistry and immunofluorescence staining were dried and fixed in 4% paraformaldehyde followed by washes in water and PBS. Sections were then placed in 0.3% hydrogen peroxide for 3 min and rinsed in distilled water. Sections were incubated in a 4% species appropriate blocking serum for 45 min. They were then incubated for 2 h with the appropriate primary antibody, diluted in PBS [anti-beta III tubulin antibody (2G10) (1:500), Abcam ab78078, Cambridge, UK; anti-beta III tubulin antibody (1:500), Abcam ab18207, Cambridge, UK; anti-AP2 alpha antibody (1:500), Abcam ab189995, Cambridge, UK; anti-AP2 alpha antibody (1:500), Abcam ab52222, Cambridge, UK; Trp1 (1:200), TYR (1:200), generous gift from Dr. V. Hearing, NCI; antiglial fibrillary acidic protein antibody, clone GA5 (1:100), Merck Millipore MAB360, Billerica, MA, USA; anti-CD31 antibody (1:100), Abcam ab28364, Cambridge, UK].

Sections undergoing immunohistochemistry were then incubated for 1 h with a species appropriate secondary antibody, diluted to a concentration of 1:200 in PBS (Vector Labs, Burlingame, CA, USA). This was followed by incubation for 30 min with horseradish peroxidase (HRP)-labeled streptavidin (VWR 95059-456, Radnor, PA, USA). Samples were developed using a Dako AEC substrate chromogen (Agilent K3464, Santa Clara, CA, USA) and then counterstained with Gill's hematoxylin. Sections were mounted with an aqueous mounting agent [SHUR/*Mount* liquid cover glass (xylene), Triangle Biomedical Sciences, INC LC-A, Durham, NC, USA].

Sections undergoing double immunofluorescence were incubated with a species appropriate, fluorescently conjugated secondary antibody diluted to a concentration of 1:200 in PBS for 1 h (Alexa Fluor conjugated secondary antibodies, ThermoFisher Scientific, Waltham, MA, USA). Sections were then treated with an Avidin-biotin blocking kit (Avidin/Biotin Blocking Kit, Vector Laboratories SP-2001, Burlingame, CA, USA) in accordance with the kit protocol. Sections were incubated with another species-appropriate 4% blocking serum in preparation for the second primary antibody for 45 min. The second primary antibody was then applied to the tissue and incubated overnight at 4°C. The following day, sections were incubated for 1 h with a species-appropriate and fluorescently conjugated secondary antibody diluted to a concentration of 1:200. Conjugated probes with different wavelengths from that of the first secondary antibody were chosen. Sections were mounted with 4′,6-diamidino-2-phenylindole (DAPI) (VECTASHIELD antifade mounting medium with DAPI, Vector Laboratories H-1200, Burlingame, CA, USA).

Images of Movat's pentachrome, van Geison, and immunohistochemistry stains were taken with Nikon Eclipse 50i and Nikon Eclipse 80i microscopes (Nikon, Melville, NY, USA). Images of immunofluorescence stains were taken with a Nikon Eclipse Ti Confocal microscope (Nikon, Melville, NY, USA). Image analysis was performed using Nikon NIS Elements (AR 3.2, Nikon, Melville, NY, USA) and/or by using a custom MATLAB (MathWorks, Natick, MA, USA) image processing script. Images were loaded into MATLAB, and a region of interest was drawn around the whole leaflet. The leaflet image was then sectioned into three portions based on the area of the tissue that corresponded to leaflet tip, middle of leaflet, and leaflet base. Using NIS Elements, thresholds were then applied based on the hue, saturation, and intensity value calculated for each protein, and the total amount of protein present was calculated for each leaflet region. Percent of each protein per leaflet region was then calculated for each leaflet overall.

### VIC *in vitro* Culture and Immunostaining

Primary human VICs were produced from AV obtained from replacement surgeries (BWH IRB protocol number 2011P001703) after informed consent was obtained. The samples were handled in Dulbecco's modified Eagle's medium (DMEM) media (Lonza, Basel, Switzerland). Endothelial cells were removed by scratching the surface with a razor blade. Cell isolation was facilitated by 1% collagenase (Sigma-Aldrich, St Louis, MO, USA) tissue digestion. The cells were expanded in DMEM (Lonza, Basel, Switzerland) supplemented with 10% fetal calf serum (FCS) (VWR, Radnor, PA, USA) until they reached 90% confluency and were passaged subsequently. VICs were used at passage 3. The cells were cultured at 37°C (5% CO_2_, 90% humidity), and all cell culture media were supplemented with 1% streptomycin/penicillin (Corning Inc., Corning, NY, USA).

For immunostaining, the cells were washed in PBS twice and fixed in 4% paraformaldehyde. The cells were incubated in a 5% species appropriate blocking serum for 1 h. They were then incubated for 2 h with the appropriate primary antibody, diluted in PBS [antibeta III tubulin antibody (2G10) (1:500), Abcam ab78078, Cambridge, UK; antiglial fibrillary acidic protein antibody, clone GA5 (1:100), Merck Millipore MAB360, Billerica, MA, USA]. Species appropriate, fluorescently conjugated secondary antibody diluted to a concentration of 1:200 in PBS for 1 h (Alexa Fluor conjugated secondary antibodies, ThermoFisher Scientific, Waltham, MA, USA) were used. Phalloidin was used to counterstain the VICs (Abcam, Cambridge, UK). The cells were imaged with a Nikon Eclipse Ti confocal microscope (Nikon, Melville, NY, USA).

### Two-Photon Imaging

Two-photon microscopy was utilized to image murine AV leaflet collagen structure and distribution by second-harmonic generation (SHG) imaging, elastin autofluorescence, and GFP-labeled melanocytes *ex vivo* (Zeiss LSM 780 NLO, SCA/E objective, Mai-Tai DeepSee HP laser, Zeiss C-Apochromat ×10/0.45 W objective). The murine hearts were fixed in 10% formalin for 24 h at 4°C and stored in PBS. Through microdissection, the AVs were cut open at the commissure between the left and right coronary leaflet and fixed on 35 mm culture dishes with superglue facilitating enface imaging of all three leaflets. PBS was added to submerge the samples. The SHG signal was generated with the Mai-Tai laser tuned to 850 nm and collection of the signal emission at 425 nm. GFP was visualized with the Mai-Tai laser tuned to an excitation wavelength of 950 nm, and the signal was collected at 525 nm. Optical sections up to 800 μm were captured in 10-μm increments into the AV leaflets and AV sinus. SHG images and GFP were overlaid for image analysis allowing for signal coregistration in intact tissues.

### ECM and Phenotypic Gene Expression Quantification From AV Leaflets

AV were obtained from wild-type C57BL/6/J, *K5-Edn3*, and *Kit* ^*Wv*/*Wv*^ mice. Tissues were collected into TRIzol (Sigma) immediately and homogenized with a needle followed by sonication on ice. NORGEN single-cell RNA purification kit (Norgen Biotek Corp., Thorold, ON, Canada) was used for RNA extraction. Complementary DNA (cDNA) was made using RevertAid First Strand cDNA Synthesis Kit (Thermo Fisher Scientific, Waltham, MA) and was found to be free of genomic DNA contamination. The levels of expression of elastin were examined via semiquantitative real-time PCR. Real-time PCR was performed using the Maxima SYBR Green/ROX qPCR Master Mix (Thermo Scientific) on a 7,500 Real-Time System (Applied Biosystems, Foster City, CA, USA). The following primers were used to assay the levels of expression of elastin: 5′-GGCTTTGGACTTTCTCCCATT and 5′-CCGGCCACAGGATTTCC. The thermal profile of the PCR reaction was 95°C for 10 min and 40 cycles of 95°C for 15 s and 58°C for 1 min. Samples were analyzed in triplicates, and the 2^−ΔΔCT^ method was used to determine relative levels of expression, which was normalized to glyceraldehyde 3-phosphate dehydrogenase (GAPDH) expression levels. The expression levels in *K5-Edn3* or *Kit* ^*Wv*/*Wv*^ mice relative to wild-type C57BL/6/J mice was estimated from the fold change calculated *via* the 2^−ΔΔCT^ method.

### Valvular Whole Tissue Proteolysis

VICs were isolated from AVs removed for severe stenosis using tissue lysis with 2% collagenase (Sigma-Aldrich, St Louis, MO, USA). Protein extraction and proteolysis were performed with the methanol-chloroform method and trypsin [(Gold Grade; Promega, Wisconsin)/RapiGest procedure (Waters, Milford, MA, USA)], respectively, as previously published (18). Fifteen micrograms of protein was used per sample. The tryptic peptides were desalted using Oasis Hlb 1cc (10 mg) columns (Waters, Milford, MA, USA), and dried with a tabletop speed vacuum (SPD1010, Thermo Fisher Scientific, USA). After resuspension in 40 μl of 5% mass spectrometry grade acetonitrile (Thermo Fisher Scientific, USA) and 5% formic acid (Sigma-Aldrich, St Louis, MO, USA), the tryptic peptide samples were analyzed by liquid chromatography–mass spectrometry.

### Mass Spectrometry and Data Processing

#### Data-Dependent Acquisition (DDA, Unbiased Peptide Sequencing)

The peptides were analyzed using the Orbitrap Fusion Lumos Tribrid mass spectrometer (Thermo Scientific) fronted with an Easy-Spray ion source, coupled to an Easy-nLC1000 HPLC pump (Thermo Scientific). The peptides were separated using a dual column set-up: an Acclaim PepMap RSLC C18 trap column, 75 μm × 20 mm, and an EASY-Spray LC heated (45°C) column, 75 μm × 250 mm (Thermo Scientific). The gradient flowrate was 300 nl/min from 5 to 21% solvent B (acetonitrile/0.1% formic acid) for 75 min, 21 to 30% solvent B for 15 min, followed by 5 min of 95% solvent B. Solvent A was 0.1% formic acid. The instrument was set to 120K resolution, and the top N precursor ions in 3 s cycle time (within a scan range of 375–1,500 m/z) were subjected to collision-induced dissociation (CID, collision energy 30%) for peptide sequencing (or MS/MS).

The MS/MS data were queried against the human UniProt database (downloaded on August 1, 2014) using the SEQUEST search algorithm, via the Proteome Discoverer (PD) Package (version 2.1, Thermo Scientific), using a 10-ppm tolerance window in the MS1 search space and a 0.6-Da fragment tolerance window for CID. Methionine oxidation was set as a variable modification, and carbamidomethylation of cysteine residues was set as fixed modification. The peptide false discovery rate (FDR) was calculated using a percolator provided by PD: the FDR was determined based on the number of MS/MS spectral hits when searched against the reverse, decoy human database. Peptides were filtered based on a 1% FDR. Peptides assigned to a given protein group, and not present in any other protein group, were considered as unique. Consequently, each protein group is represented by a single master protein (PD grouping feature).

#### Parallel Reaction Monitoring (PRM, Targeted Peptide Sequencing)—Spectral Library

The peptide library resources for glial fibrillary acidic protein (GFAP) and TUJ1 included the DDA data above (both proteins), our previous AV proteomics data (GFAP) (14), and http://www.peptideatlas.org/ [TUJ1 (TUBB3)]. We reacquired two peptide spectra per protein running the instrument in a targeted mode, also known as PRM (19, 20). The chromatographic gradients were the same as the DDA runs. For each precursor peptide mass (below), the isolation window was 1 m/z, and we alternated between CID and HCD (higher collision energy dissociation, 30% collision energy) for sequencing. CID and HCD fragments were scanned in the Orbitrap at 30K resolution within a scan range of 100–1,000 m/z. Using higher resolution and quality PRM scans, the following peptides were confirmed in the valve tissue: TUBB3_ISVYYNEASSHK (M + 3H)^3+^ = 466.561, retention time (RT) of 22 min; TUBB3_LHFF(Mox)PGFAPLTSR (M + 2H)^2+^ = 810.921, RT of 76 min; GFAP_ALAAELNQLR (M+2H)^2+^ = 549.817, RT of 45 min; and GFAP_LEVERDNLAQDLATVR (M+3H)^3+^ = 614.663, RT of 54 min (Supplementary Figure 1).

### Western Blotting Analysis

A human AV sample was pulverized in liquid nitrogen and resuspended in radioimmunoprecipitation assay (RIPA) buffer (Thermo Fisher Scientific, USA) with 1% protease inhibitor cocktail (Roche, Switzerland). The protein was precipitated and isolated in 6 M urea/2 M thiourea/100 mM TEAB (pH 8.0). Protein concentration was measured using the Pierce 660nm Protein Assay (Bradford) method (Thermo Fisher Scientific, USA). Human cortical protein (Takara Bio, Mountain View, CA, USA) and the AV protein were denatured and boiled in Laemmli buffer (Bio-Rad, Hercules, CA, USA). Total protein was separated by 10% sodium dodecyl sulfate–polyacrylamide gel electrophoresis (SDS-PAGE) and transferred using the iBlot Western blotting system (Life Technologies, Carlsbad, CA, USA). Primary antibodies against TUJ-1 (1:500; Abcam, USA) and GFAP (1:5,000; EMD Millipore, Billerica, MA, USA) were used. Protein expression was detected using Pierce ECL Western Blotting substrate reagent (Thermo Fisher Scientific, USA) and ImageQuant LAS 4000 (GE Healthcare, Arlington Heights, IL, USA).

## Results

### Valve Melanocytes Exhibit Neuronal and Glial Phenotypic Markers

**Figure 1:**
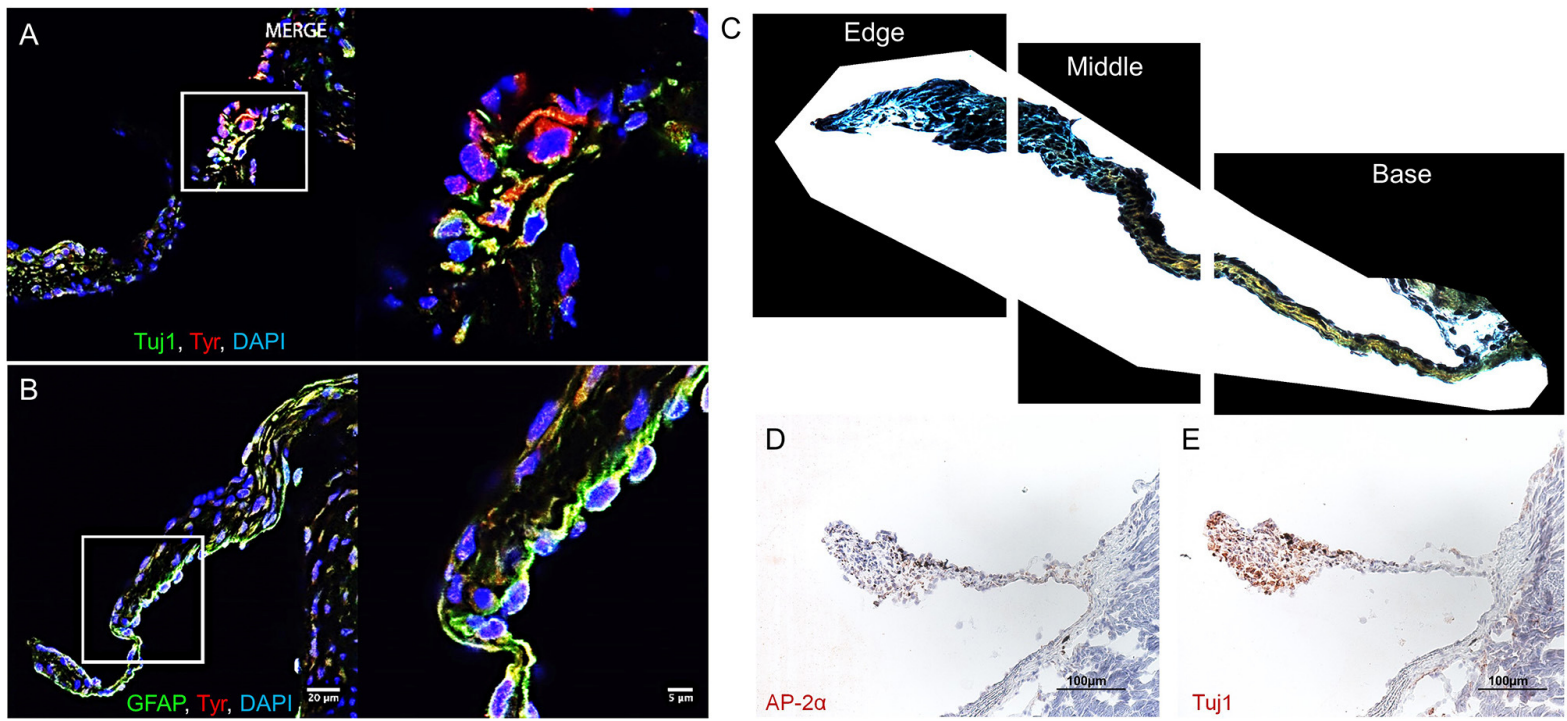
Aortic valve (AV) interstitial cell (VIC) phenotypes. Melanocytic, neuronal, and glial proteins in mouse AV leaflets. Immunofluorescence on mouse AV tissue sections for **(A)** the neuronal marker tubulin beta 3 class III (Tuj1), tyrosinase (Tyr), and cell nuclei (DAPI); **(B)** the glial marker glial fibrillary acidic protein (GFAP), Tyr, and DAPI. Scale = 20 μm for large and 5 μm for magnified immunofluorescence images. **(C)** Representative image of leaflet sectioned into thirds by area using a custom image analysis script. **(D)** Immunohistochemistry of the glial marker AP-2α and **(E)** Tuj1. Scale = 100 μm for immunohistochemistry images.

In C57BL/6 murine AV leaflets, a subpopulation of melanocytic VICs immunopositive for tyrosinase (Tyr) (Figure 1A) expressed the common neuronal lineage marker neuron-specific class III beta-tubulin (Tuj1). A separate melanocytic VIC subpopulation immunopositive for tyrosinase (Figure 1B) expressed the common glial intermediate filament GFAP. To assess the regional distribution and ECM associations between these melanocytic subpopulations, Movat's pentachrome and Elastica van Gieson stained ECM components in histological sections of C57BL/6 mouse AV leaflets, and immunohistochemistry identified the presence of neuronal and glial markers in adjacent histological sections (*N* = 3 AVs; 18 sections imaged per AV). After manually selecting the perimeter of each leaflet section, a custom image analysis script segmented the leaflet into equal thirds by area (Figure 1C). Whereas VICs positive for the transcription factor AP-2α, commonly expressed in neurons and glia, appear distributed throughout the leaflets (Figure 1D), qualitative analyses indicated predominance of Tuj1-positive VICs at the free edge of the leaflets (Figure 1E).

**Figure 2:**
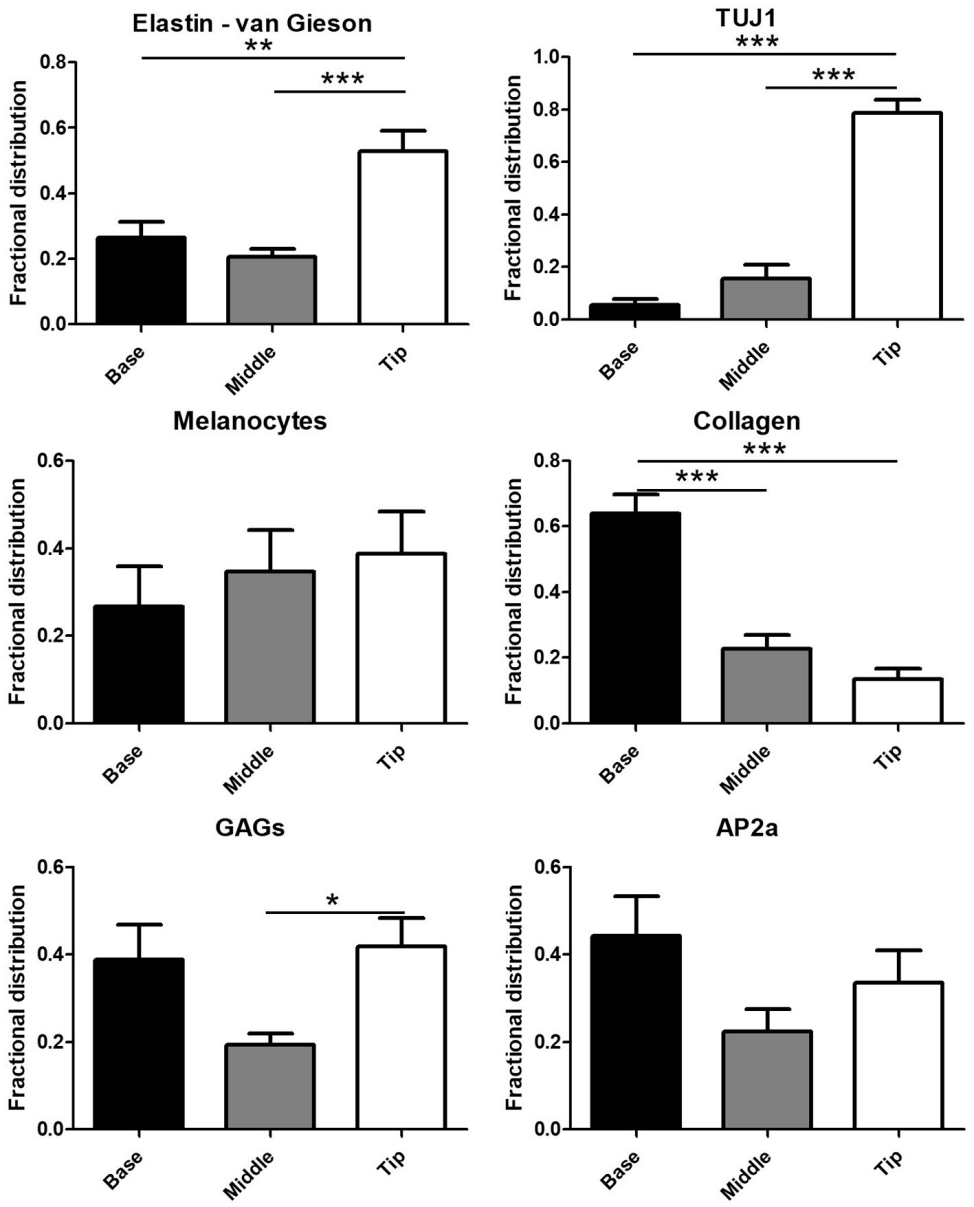
Quantification of the glial transcription factor AP-2α, Tuj1, collagen, glycosaminoglycans (GAGs), elastin, and melanin in digitally segmented leaflet portions (data represent fractional distribution of left coronary, noncoronary, and right coronary leaflets of *n* = 3 wild-type mice). *p < 0.05, **p < 0.01, ***p < 0.001.

Automated quantification of the Movat's stain in each of the three image portions showed 76.9 ± 17.0% of collagen in the base portion of the leaflets (Figure 2) with the remaining 23.1% distributed between the middle portion (21.7 ± 12.7%) and leaflet tip (1.3 ± 2.9%). GAGs exhibited a more symmetric distribution with 38.8 ± 23.8% in the leaflet base, 19.3 ± 11.3% in the middle of the leaflets, and 41.8 ± 20.1% at the tip of the leaflets. A majority of van Gieson stained elastic fibers localized to the tip of the leaflets (52.9 ± 19.1%) with 26.4 ± 15.3% in the leaflet base and 20.7 ± 7.0% in the middle third of the leaflets. Similar to elastin, the Tuj1-positive VICs localized largely to the tip of the leaflets (78.8 ± 15.0%). A smaller portion of Tuj1-positive VICs were observed in the middle third (15.6 ± 15.7%) and base (5.6 ± 6.2%) of the leaflets (Figure 1C). Glial-like VICs were identified by immunostaining for the transcription factor AP-2α. The AP-2α-positive VICs exhibit a distribution similar to that of GAGs with 44.2 ± 27.1% of the stain at the base of the leaflets, 22.3 ± 15.4% in the middle third of the leaflets, and 33.5 ± 22.5% at the leaflet tip (Figure 2).

**Figure 3:**
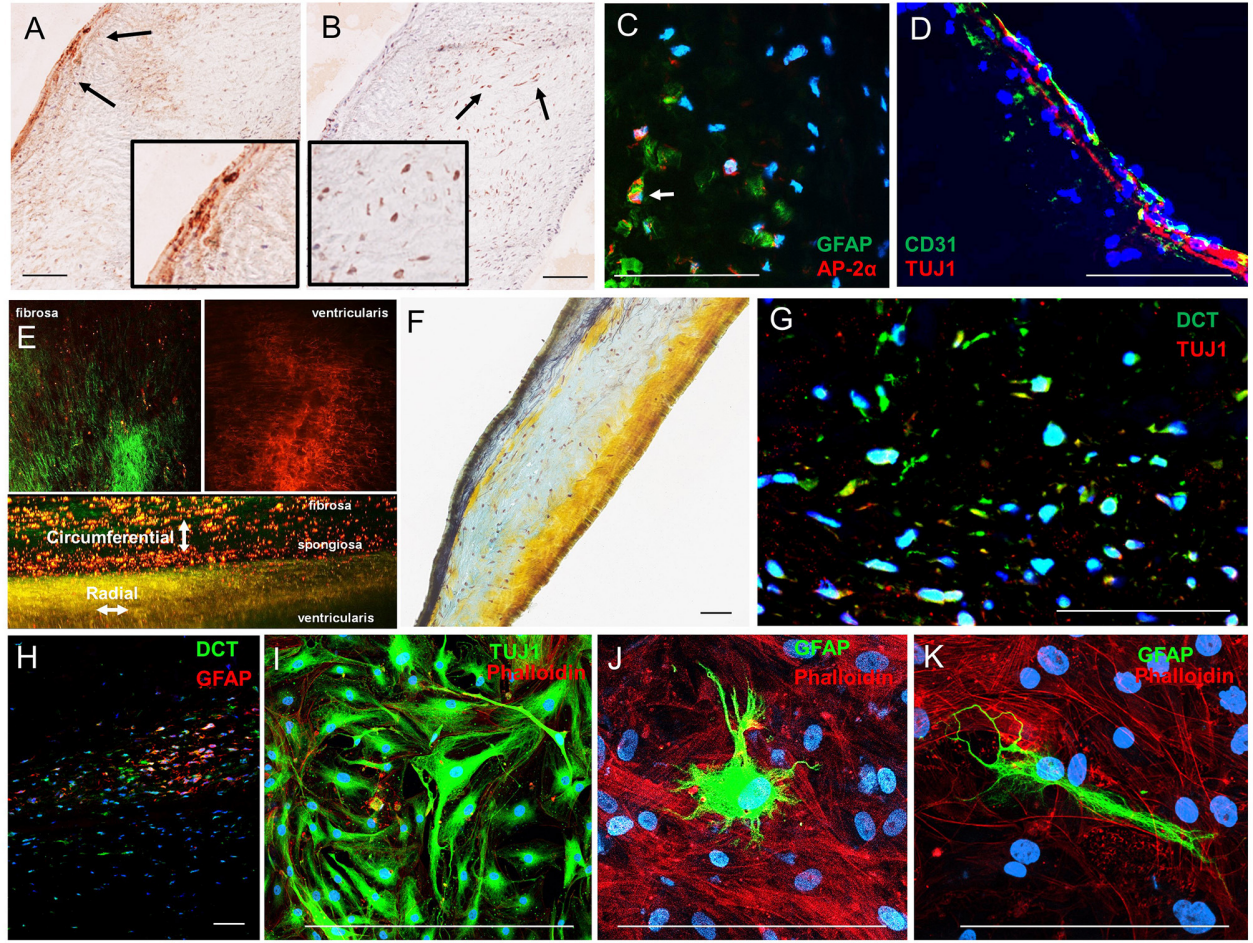
Analysis of VIC phenotypes in human AV tissues. **(A)** Immunohistochemistry of neuronal (TUJ1) and **(B)** glial (AP-2α) in sections of a human AV leaflet. **(C)** Immunofluorescence of the glial markers GFAP (green) and AP-2α (red) in a histological section of a human AV leaflet. **(D)** Immunofluorescence of the endothelial marker CD31 (green) and the neuronal marker Tuj1 (red) in a histological section of a human AV leaflet. **(E)** Two-photon imaging of an intact human AV leaflet with second harmonic generation identified collagen fibers (green) and cellular and elastin autofluorescence (red). **(F)** Movat's pentachrome staining of a human AV leaflet histological section with collagen fibers (yellow), GAGs (blue), and elastin (black). **(G)** Immunofluorescence of the melanocytic marker dopachrome tautomerase (DCT, green) and a neuronal marker (TUJ1, red) in a histological section of a human AV leaflet. **(H)** Immunofluorescence of the melanocytic marker dopachrome tautomerase (DCT, green) and a glial marker (GFAP, red) in a histological section of a human AV leaflet. **(I)** Immunofluorescence of the neuronal marker (TUJ1, green) and the actin cytoskeleton (red) in human VICs in culture. **(J, K)** Immunofluorescence of the glial marker (GFAP, green) and the actin cytoskeleton (red) in human VICs in culture. Scale = 100 μm.

Immunohistochemistry analyses of human AV histological sections showed Tuj1-positive VICs localized adjacent to the ventricularis face of the leaflets (Figure 3A), consistent with previous observations of subendothelial neurons in porcine AV leaflets (13). AP-2α-positive VICs localized to the spongiosa region of the human leaflets (Figure 3B). Double immunofluorescence staining of the leaflets confirmed that AP-2α-positive VICs coexpress GFAP (Figure 3C), and Tuj1-positive VICs localize directly adjacent to the ventricularis endothelium (Figure 3D). Two-photon imaging (Figure 3E) and Movat pentrochrome staining of histological sections (Figure 3F) showed the characteristic trilaminar leaflet structure in the human AV leaflets used in the present study, with circumferentially oriented collagen fibers in the fibrosa, a GAG-rich spongiosa layer, and radially oriented elastic fibers in the ventricularis. The AP-2α/GFAP VIC localization in the GAG-rich spongiosa and Tuj1 VIC localization in the elastin-rich ventricularis of the human AV leaflets (Figures 3A,B) is consistent with the VIC–ECM associations observed in the mouse AV leaflets (Figure 2).

Human VICs do not express TYR nor produce melanin pigment; however, populations of VICs exhibited expression of the melanocytic marker dopachrome tautomerase (DCT, also known as tyrosinase-related protein-2) as identified by immunohistochemistry (Supplementary Figure 2A) and immunofluorescence (Supplementary Figure 2B) in histological sections of human AVs. These VICs exhibit similar localization patterns to the Tuj1- and GFAP-positive VICs described above, and coimmunofluorescence revealed coexpression of these markers in DCT-positive VIC populations (Figures 3G,H). VICs immunopositive for Tuj1 or GFAP were also observed in cultures derived from human AV leaflets (Figures 3I–K).

**Figure 4:**
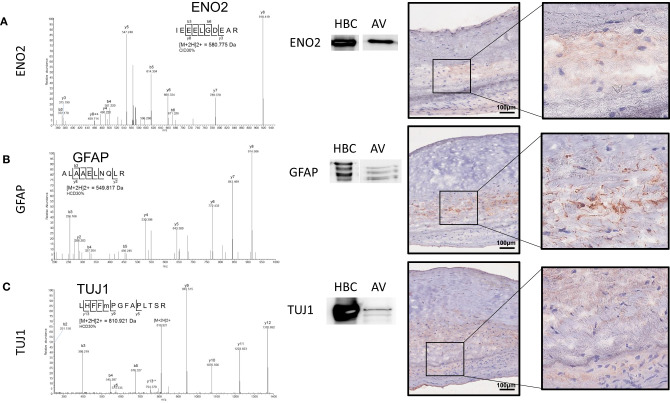
Parallel reaction monitoring proteomics and western blotting on human AV tissue protein lysate and human AV tissue sections for **(A)** ENO2, **(B)** GFAP, and **(C)** TUJ1; Western blotting for each protein [HBC (human brain cortex)] positive control, AV (aortic valve protein). Immunohistochemistry on human AV leaflet sections for each protein. Scale = 100 μm.

We recently mapped the proteome of the human AV and established the molecular signatures of the fibrosa, spongiosa, and ventricularis layers (14). Similar to the immunohistochemistry data presented above, GFAP peptides were specific to and abundant within the spongiosa layer. In the current study, we employed unbiased proteomics for human AV tissue screening and targeted proteomics, parallel reaction monitoring (PRM) technology (20). These analyses confirmed the presence of neuronal and glial markers. Specifically, enolase 2 (Eno2), GFAP, and Tuj1 were observed in the human AV (Figures 4A–C). Representative peptide MS/MS spectrum acquired using PRM for GFAP and Tuj1 verified the existence of these proteins in the human AV tissue. Western blotting supported the mass spectrometry data, showing protein bands immunopositive for GFAP and Tuj1 in AV leaflets similar to those observed in lysates from human brain cortex (HBC, Figures 4A–C).

### Melanocytes Regulate Elastin in AV Leaflets

**Figure 5:**
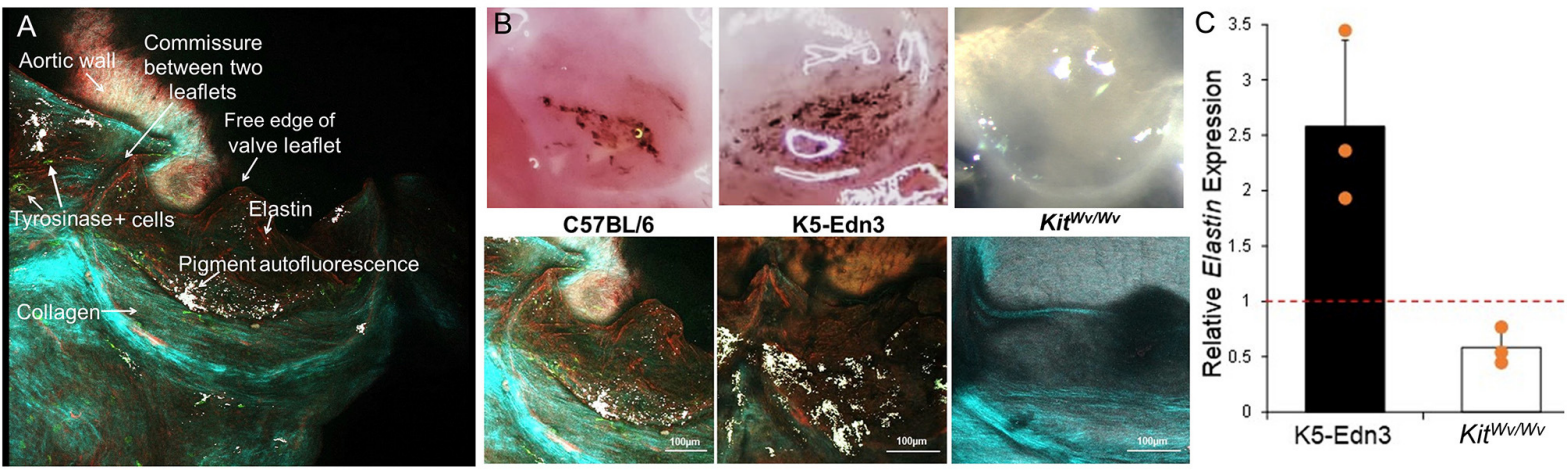
ECM analyses in mice with altered melanocytic populations. **(A)** *En face* imaging of a mouse AV leaflet with GFP-labeled Tyr+ cells (green) using two-photon microscopy. Second harmonic generation identifies collagen fibers (light blue/green) and autofluorescence associates with elastin (red) and melanin pigment (white). **(B)** Top panel: photographs under a dissection microscope to demonstrate relative amounts of pigment in AV leaflets from C57BL/6, K5-Edn3, and Kit ^Wv/Wv^ mice. Bottom panel: two-photon imaging of second harmonic generation identified collagen fibers (light blue/green) and elastin (red) and melanin pigment (white) autofluorescence in AV leaflets from C57BL/6, K5-Edn3, and *Kit* ^*Wv*/*Wv*^ mice. **(C)** qPCR analysis of *elastin* gene expression from AV leaflets from K5-Edn3 and *Kit* ^*Wv*/*Wv*^ mice (*N* = 3 per group) normalized to C57BL/6 gene expression (dotted line).

The data above show specific localization patterns between melanocytic VIC populations and leaflet ECM components. To determine the potential role of these VICs in ECM patterning, we employed mouse models with altered melanocytic populations. Hearts of TYR-CreERT2/ mT/mG mice that were induced with 4HT to identify tyrosinase-positive melanocytes were collected and subjected to two-photon imaging (*N* = 3). GFP-positive cells (TYR positive) were observed throughout the mouse AV leaflets (Figure 5A). Most of the GFP-positive cells were in close proximity to auto-fluorescent melanin (white signal), which was observed to overlap with pigmented regions on the leaflets during tissue examination. SHG collagen imaging and elastin auto-fluorescence showed circumferentially aligned collagen fibers (cyan) and radially aligned elastic fibers (red) (Figure 5A). To assess potential relationships between melanocytic activity and AV patterning, we employed hyperpigmented (K5-Edn3) and hypopigmented (*Kit*^*Wv*/*w*^) mice. Bright field images of representative AV leaflets demonstrate the pigmentation differences in these mice (Figure 5B, top panel). Two-photon imaging revealed increased, disorganized elastic fibers in hyperpigmented K5-Edn3 mice and a complete loss of elastic fibers in the AV of hypopigmented *Kit*^*Wv*/*Wv*^ mice compared to C57BL/6 controls (Figure 5B, bottom panel). Gene expression analysis corroborated these findings, revealing a 42% decrease in elastin expression in *Kit*^*Wv*/*Wv*^ mice (*N* = 3) and a 2.6-fold increase in elastin expression in K5-Edn3 mice (*N* = 3) (Figure 5C).

## Discussion

In mouse AV, melanocytes expressed either neuronal or glial markers. Similar VIC populations were observed in human AV leaflets, including the expression of the melanocytic marker DCT. Compared to wild-type mice, mouse mutants with increased cardiovascular melanocytes or no cardiovascular melanocytes exhibit increased and diminished elastin, respectively. Given the importance of elastin in AV homeostasis and disease (21, 22), the role of melanocytic VICs in elastin regulation warrants further investigation. While the data demonstrate a novel relationship between melanocytic populations and elastin regulation in aortic valve leaflets, causal relationships remain unclear. Future studies are needed to determine the mechanisms associated with these observations and the relevance to aortic valve development and disease. The present data indicate that melanocytic VICs participate in elastin generation in mouse AV, and these cells may be of interest for future therapeutic targets and tissue engineering seeking to induce elastogenesis in human AV.

In human embryonic AV, the first elastic fibers are observed at 7 weeks of gestation (23). This time point corresponds to approximately mouse developmental stage E14.5, the time at which melanocytes first appear in murine cardiac valves (24). This developmental period also corresponds to a change in cardiovascular biomechanics due to an increase in blood pressure and a rapid rise in heart rate, suggesting that elastic fibers allow the heart to adapt to the increased mechanical stresses (23). Correspondingly, species with low pressure circulatory systems (e.g., two-chambered hearts) often do not exhibit elastic fibers in cardiovascular tissues. Of note, species with two-chambered hearts are also devoid of identifiable cardiovascular melanocytes as identified by DCT expression (24). These observations provide circumstantial support to the hypothesis that these mechanisms may be conserved across species, and cells similar to the mouse melanocyte VICs may also control elastogenesis in human AV. In addition, the noted correlation between the appearance of melanocytic populations and sudden changes in developmental blood pressure suggests that these cells could be mechanosensitive. Elastic fibers store energy as the AV leaflets stretch radially to seal the valve during diastole. Alignment of Tuj1-positive melanocytic cells along this direction of maximum tensile loading may sense the changing mechanical environment during development and direct the proper elastic fiber alignment. In mouse tissues, pigmented regions are stiffer than nonpigmented regions of AV leaflets, suggesting that the melanocytic cells may play an additional role in the unique demands of mouse AV biomechanics (e.g., heart rates an order of magnitude higher than those in humans).

Future studies are needed to assess the relevance and differences between the VICs exhibiting neuronal vs. glial phenotypic markers and the association of these novel cell phenotypes with elastin synthesis in both mouse and human AV development. We have observed Tuj1- and GFAP-positive cells that do not seem to express melanocytic markers. Qualitatively, however, it seems that most of the Tuj1- and GFAP-positive cells coexpress the melanocytic markers. Currently, we are not certain whether these are distinct populations or if the lack of markers in some cells is an artifact/incomplete labeling in the immunofluorescence. Future studies are needed to more carefully isolate and characterize these cellular populations. Although we observe colocalization between the Tuj1-positive melanocytic cells and elastic fibers, we cannot rule out a role for the GFAP-positive cells in elastin regulation. Our genetically modified mice alter melanocytic populations (not specifically Tuj1- or GFAP-positive populations). Therefore, it is possible that the melanocytic GFAP-positive populations are involved in elastin regulation—perhaps through a paracrine mechanism—even though they do not localize to these regions. Similarly, although they localize in regions with elastic fibers, it is unclear whether the Tuj1-positive melanocytic VICs directly contribute to elastic fiber deposition or if these cells contribute to elastogenesis through paracrine signaling with other VIC populations. Given the lack of association between neuronal and glial phenotypes and elastin synthesis in other tissues, it is possible that these populations could direct elastic fiber formation through fibroblastic and/or smooth muscle cell populations that reside in the valve leaflets. Dermal melanocytic precursors, however, have been shown to express elastin binding protein, and an elastin-derived peptide stimulates melanogenesis and dendrite formation (25). However, to this point, the source of elastin and the exact role of melanocytic populations in patterning heart valves remain unknown. Regardless of their exact contribution to the mechanisms of elastic fiber deposition in developing AV, these cells warrant consideration in therapeutic strategies seeking to regenerate native AV ECM *in situ* or design of tissue engineered valve replacements.

## Data Availability Statement

The mass spectrometry proteomics data have been deposited to the ProteomeXchange Consortium via the PRIDE (26) partner repository with the dataset identifier PXD025872.

## Ethics Statement

The animal study was reviewed and approved by Florida International University IACUC. AV leaflets were collected from AV replacement surgeries for severe AV stenosis [Brigham and Women's Hospital (BWH) IRB protocol number 2011P001703]. Non-diseased AVs were obtained from autopsy (BWH IRB 2014P001505).

## Author Contributions

JH, FS, MC, LK, and EA developed the original idea for the study. JH, FS, MC, and XL performed the experiments, analyzed the data, and drafted the manuscript. TP helped perform mass spectrometry and imaging experiments, and associated data analyses. PV helped procure and analyze data from human aortic valve tissues and cells. HH and SS helped perform the proteomics analyses and analyzed the resulting data. SB helped procure human aortic valve tissue for analyses and data interpretation. MA aided in experimental design and data interpretation. All authors contributed to the article and approved the submitted version.

## Conflict of Interest

The authors declare that the research was conducted in the absence of any commercial or financial relationships that could be construed as a potential conflict of interest.
