# Supplementary material for: Elastogenesis Correlates With Pigment Production in Murine Aortic Valve Leaflets
Source: Front Cardiovasc Med. 2021 Jun 22;8:678401. doi: 10.3389/fcvm.2021.678401 (PMC8257952; doi:10.3389/fcvm.2021.678401)
Supplement: Supplementary file 1 [file Presentation_1.PPTX]

## Slide 1
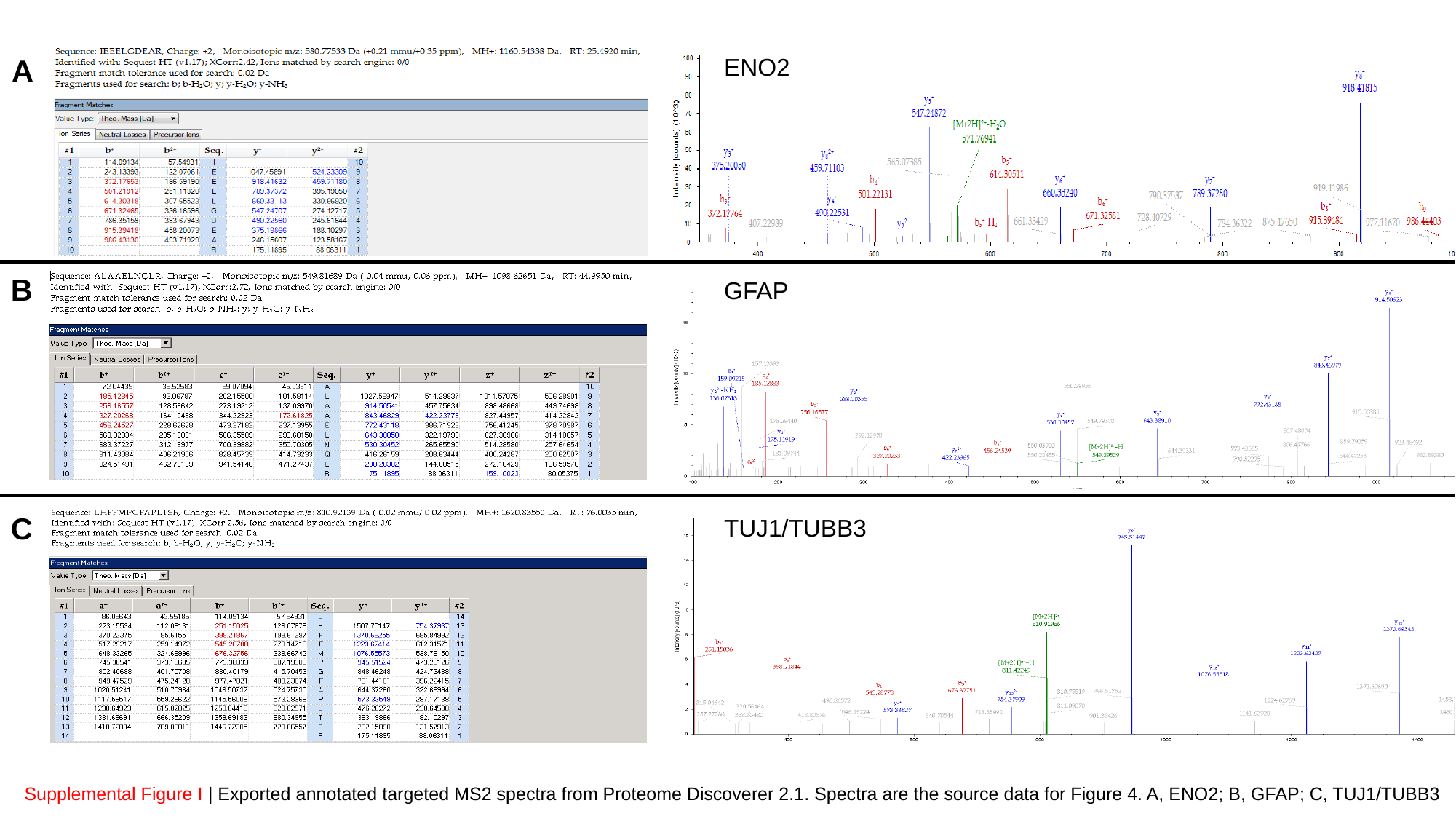

A
ENO2
B
GFAP
C
TUJ1/TUBB3
Supplemental Figure I | Exported annotated targeted MS2 spectra from Proteome Discoverer 2.1. Spectra are the source data for Figure 4. A, ENO2; B, GFAP; C, TUJ1/TUBB3

## Slide 2
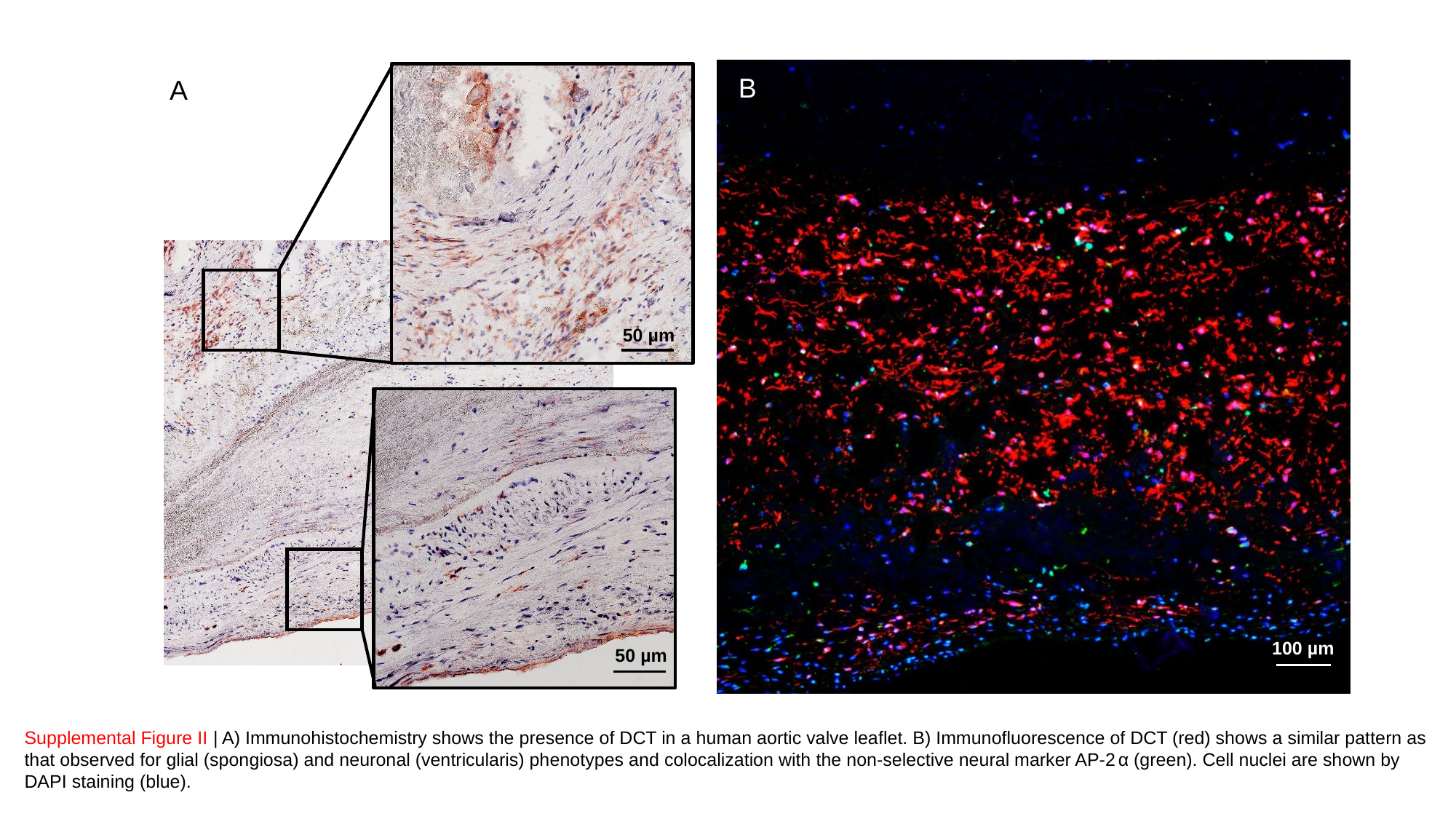

B
A
50 µm
100 µm
50 µm
Supplemental Figure II | A) Immunohistochemistry shows the presence of DCT in a human aortic valve leaflet. B) Immunofluorescence of DCT (red) shows a similar pattern as that observed for glial (spongiosa) and neuronal (ventricularis) phenotypes and colocalization with the non-selective neural marker AP-2α (green). Cell nuclei are shown by DAPI staining (blue).
